# The Depths of Elder Abuse: A Narrative Review with Medico-Legal Perspectives

**DOI:** 10.3390/bs16020180

**Published:** 2026-01-27

**Authors:** Ruben Nițulescu, Andreea Calapod, Laura Tribus, Sorin Hostiuc

**Affiliations:** 1 Department of Internal Medicine, Carol Davila University of Medicine and Pharmacy, 020021 Bucharest, Romania; 2 Ilfov County Emergency Clinical Hospital, 022104 Bucharest, Romania; 3 Department of Legal Medicine and Bioethics, Carol Davila University of Medicine and Pharmacy, 020021 Bucharest, Romania; 4 National Institute of Legal Medicine, 042122 Bucharest, Romania

**Keywords:** elder abuse, aging, risk factors, neglect, forensic medicine

## Abstract

Elder abuse is an increasingly common problem in modern society, in the context of rapid population aging. Despite increasing awareness, this phenomenon remains heavily underreported, and effective interventions are yet to be made, thus leading to significant medical, social, and legal implications. The purpose of this review is to present an updated situation of the depths of elder abuse, presenting its prevalence both at the global and European level, the two main environments in which it is the most common (community and institutional settings), different forms of abuse, risk factors, and consequences for each one of them, as well as medico-legal aspects on the matter. A narrative review was conducted based on PubMed/MEDLINE, Scopus, and Web of Science databases, in association with data presented in reports from international organizations. The review included only articles published in English, in peer-reviewed journals, addressing elder abuse in adults aged 60 years and older, and those that didn’t respect the criteria were excluded. Elder abuse comes in different forms, most of the time overlapping, with psychological abuse being the most prevalent. Each one of them has its own risk factors and specific consequences, but all of them will eventually lead to increased morbidity, accelerated cognitive impairment, and functional decline. In community settings, the elders usually experience abuse related to dependency on the family and social isolation, while in institutional settings, abuse is frequently associated with understaffing and inadequate care. From a forensic perspective, functional and cognitive decline complicate the proper documentation of the abuse. Thus, the role of the physician in providing legal support to the victim is essential. Elder abuse continues to be heavily overlooked, losing sight of the fact that its consequences extend beyond immediate physical harm, affecting the general physical and mental health of the victims. A possible solution to this problem is envisioned, with the purpose of raising awareness of this situation and contributing to a change in the perspective from which society looks at the elderly.

## 1. Introduction

The world is experiencing a rapidly increasing demographic trend known as population aging, which poses serious problems for many facets of society. According to projections, there will be over two billion people over 60 by 2050, making up almost 25% of the world’s population (World Health Organization (WHO, 2015)). Numerous economic, social, and cultural developments are associated with this demographic shift, as is an increase in the vulnerability of the elderly population. Elder abuse is one of the most alarming and often unreported forms of violence. “A single or repeated act or the failure to take appropriate action that takes place within a trusted relationship and causes an older person to suffer or be harmed” is what the World Health Organization (WHO) defines as elder abuse (WHO, 2024). Financial exploitation, sexual abuse, emotional, psychological, or physical abuse, as well as active and passive neglect, are some of the ways that this problem can appear. Each type of abuse carries significant repercussions for the physical, mental, and social well-being of the older individual as well as legal and ethical implications for the offenders and the organizations involved. The community’s overall elder abuse rate, according to recent research, is approximately 15.7%; however, because many incidents are concealed, the actual numbers may be much higher (Yon et al., 2017). Various national studies came in support of this concern. A large national study from Australia reported that approximately 1 in 6 older adults had experienced at least one form of abuse (Qu et al., 2021). The prevalence may be higher than 60% in care facilities like nursing homes, particularly in cases of psychological abuse and neglect (Cooper et al., 2008). Because victims are dependent on their abuser, feel ashamed, fear losing their social or financial support, or do not have access to an efficient reporting and intervention system, many cases remain unreported (Roberto, 2016).

Abuse can have many causes, including relationship dynamics (family conflict, unequal power), community influences (social exclusion, lack of support), societal issues (discrimination against older adults, legal flaws), and individual factors (deteriorating cognitive abilities, pre-existing health conditions, dependence on others for physical needs) (Pillemer et al., 2016; Storey, 2020). In family settings, elder abuse may also be driven by inheritance impatience, with relatives seeking premature control over assets through coercive pressure (Joint Select Committee on Coercive Control, 2021). From a legal perspective, elder abuse can be difficult to detect due to the lack of training among professionals and the low level of awareness among the public (Dong, 2015). The role of physicians is not only in providing the appropriate medical care, but also in detecting and reporting the abuse, regardless of the form it takes. Thus, the purpose of this review is to present different forms of abuse, risk factors that may lead to them, consequences of abuse, and also forensic aspects of elder abuse, to provide a better understanding of the matter and to suggest possible solutions.

## 2. Methods

### 2.1. Study Design and Literature Search Strategy

The article represents a narrative review of the literature addressing the depths of elder abuse, which focuses on its prevalence, different forms, risk factors, consequences, as well as the medico-legal aspects implied. The aim of the review was to also present both the similarities and differences between the two main settings in which elder abuse may occur: community settings and institutional settings.

The main sources of information were the following electronic databases: PubMed/MEDLINE, Web of Science, and Scopus. Additional sources included reports from the World Health Organization (WHO), European Union bodies, and other national research institutions. The search strategy combined both MeSH and free-text terms. Core concepts included terms related to “elder abuse”, while additional studies addressing specific forms of abuse, prevalence, risk factors, community and institutional settings, consequences of abuse, and forensic aspects were identified through complementary search terms and full-text screening.

### 2.2. Inclusion/Exclusion Criteria and Study Selection

Studies were included if they addressed elder abuse (persons aged 60 and above), were written in English, and were published in peer-reviewed journals or as official institutional reports. Studies focusing on abuse without age-specific analysis, and case reports with no analytical relevance were excluded.

The studies were selected after a brief screening of titles and abstracts for relevance, followed by a comprehensive full-text assessment.

In total, 43 publications met the inclusion criteria and were included in this narrative review. Approximately 10 studies focused on different forms of abuse, including physical, psychological, financial, and sexual abuse, as well as neglect. Several publications addressed more than one form of abuse, resulting in overlap across categories. The remaining studies addressed prevalence, risk factors, consequences, and medico-legal aspects of elder abuse.

## 3. Results

### 3.1. Forms of Elderly Abuse

Elder abuse may take different forms—physical, psychological, financial, sexual, or neglect (Table 1). Each of these forms has its characteristics, risk factors, and consequences. The main similarity between them is that all of them will eventually lead to the alteration of the health and mental status of the victim (Dong, 2015; Lachs & Pillemer, 2015).

One form of elder abuse is represented by physical abuse. This kind of abuse consists of using violence to provoke pain, injuries, or suffering upon an older person. Most of the time, it includes slapping, hitting, pushing, shoving, or unjustified immobilization of the elder. Its results are also easy to recognize, consisting of bruises, fractures, abrasions, lacerations, or burns (Dong, 2015; Lachs & Pillemer, 2015). One should keep in mind that these manifestations can also be caused accidentally, due to the biological fragility of the elderly, meaning that not all trauma is the result of intentional abuse. However, results of conducted studies suggest that physical abuse is more likely to be the cause of bruises on unusual areas (e.g., face, neck, inner thigh, posterior chest) than accidental injuries (Lachs & Pillemer, 2015). A correct assessment of clinical history and behavior of the victim is essential, given the fact that fractures can range from rib fractures to traumatic brain injury.

Even though psychological abuse is a very common form of abuse, it also tends to be very underestimated (Lachs & Pillemer, 2015). Some relevant examples include insults, shouting, ignoring, or restricted social contacts (Roberto, 2016). Even though it doesn’t involve visible trauma, psychological abuse can have a big impact on the mental health of the elderly, resulting in low self-esteem, anxiety, or even depression (Wiglesworth et al., 2010). Psychological abuse, including gaslighting and coercive control, frequently co-occurs with financial exploitation (Lachs & Pillemer, 2015). Emotional manipulation can negatively affect an older person’s confidence and judgment, which may, in turn, facilitate inappropriate influence over financial matters (Jackson & Hafemeister, 2013a).

Financial abuse is another form of abuse, and consists of stealing money, selling properties without consent, and using various illegal methods to forge a signature or gain some material goods. Financial abuse of an elder is defined as using their financial resources or assets in an illegal, unethical, or unauthorized way (Jackson & Hafemeister, 2013a). Financial abuse can go undetected for a long time, most of the time due to the dependency on loved ones or the cognitive decline of the elder. In many cases, financial abuse does not occur in isolation, but in association with psychological abuse, including gaslighting, intimidation, and coercive control (Dong, 2015). The Australian National Elder Abuse Prevalence Study indicates that adult children of the older person are the most common offenders of elder abuse overall, with psychological, financial, and physical abuse being the main forms of abuse (Qu et al., 2021). Moreover, Australian research suggests that sons are more frequently identified as perpetrators than daughters (Kaspiew et al., 2016).

Although it can take an active shape (intentional), neglect is one of the few forms of abuse with a passive component (due to lack of resources or knowledge). Neglect can be present either in families with limited resources or in institutional settings, and it involves the failure to provide the necessary care for an elder—nutrition, medical treatment, hygiene, or supervision. However, neglect is frequently characterized not only by the omission of basic care, but also by its normalization, both within community settings and caregiving environments (Goiran, 2018). Its results consist of malnutrition, dehydration, decubitus ulcers, infections, and can even lead to death (Lachs & Pillemer, 2015). A report published by Yon et al. states that neglect represents the most reported form of abuse in institutional settings, with up to 64.2% of residents being affected by it (Yon et al., 2019).

Last but not least, sexual abuse represents a form of abuse with severe implications for the elderly. Sexual abuse of the elderly tends to be overlooked. It refers not only to any forced sexual act, but also to non-consensual touching, sexual remarks, or intentional exposure to sexual material. It can sometimes be objectified through bruises, abrasions, and lacerations in the anogenital area or abdomen, and can even cause UTIs. It is rarely reported, mostly because of shame or fear, studies suggesting that less than 1% of sexual abuse cases are being reported (Bows, 2018; Teaster & Roberto, 2004).

### 3.2. Prevalence

The prevalence of elder abuse depends not only on the type of abuse but also on the region. Although it represents a global issue, the difference in mentality around the globe may lead to underreporting and even differences in the definition of “abuse” (Cooper et al., 2008).

In terms of global prevalence, a large meta-analysis by Yon et al. (which included 52 studies from 28 countries), held in community settings, states that about 15.7% of elders had experienced at least a form of abuse in 2017 (Yon et al., 2017). The distribution of different types of abuse, as presented in the study: Physical abuse, 6.8%; Psychological abuse, 11.6%; Financial abuse, 6.8%; Neglect, 4.2%; Sexual abuse, 0.9% (Yon et al., 2017). Other studies also depict psychological abuse as the most common form of abuse in community settings. A study conducted by K.A. Roberto in 2016 estimated that approximately 11.6% of elders experienced emotional abuse, with those living alone and women being more prone to it (Roberto, 2016). One aspect that should be considered is the fact that these data come from self-reported studies, which means that there is a risk of underreporting. Different reasons for hiding this kind of abuse may include fear of stigma, dependency on the aggressor (usually even a family member), especially in countries with strong traditional values. The prevalence in institutional settings (nursing homes) is higher (Table 2). Another Yon et al. study, held in 2019, suggests that about 64% of staff from this kind of institutional setting admit to having committed at least one form of abuse in the past 12 months (Yon et al., 2019). Even though the studies didn’t have enough data to calculate an overall self-reporting data prevalence, the distribution of different types of abuse, as estimated in the study, is: Physical abuse, 14.1%; Psychological abuse, 33.4%; Financial abuse, 13.8%; Neglect, 11.6%; Sexual abuse, 1.9% (Yon et al., 2019). While the prevalence is obviously higher in institutional settings, it is also important to mention that underreporting should also be considered, due to the total dependence of the elders on the staff.

When it comes to prevalence in Europe, despite the fact that data on elder abuse at the European level are fragmentary, general trends follow the global pattern. A systematic review conducted by WHO Europe states that the prevalence of elder abuse in the community is supposed to be between 10% and 30%, depending on the country (Sethi et al., 2011). Some countries (such as Germany, Sweden, and the Netherlands) have developed efficient monitoring systems, allowing them to gather more accurate data. However, in other countries, especially from Eastern Europe (including Romania), official data are limited. However, a prevalence of over 20% in terms of psychological abuse has been reported in some regional studies, especially among elders living with relatives in multigenerational households (Pillemer et al., 2016). The prevalence in European institutional settings is estimated to be between 20% and 50%, depending on the type of abuse and the country. Unfortunately, a lack of personnel and bureaucratic pressure contributes to the tolerance of abuse (Cooper et al., 2008). The European Union is aware of the situation and has repeatedly addressed the need to implement policies to protect the elderly, but there is still no mechanism for collecting data on abuse. However, programs like Eurostat and Age Platform Europe are trying to fill these gaps through joint research and standardizing the definitions of abuse (A world without elder abuse requires systemic action!—Elder Abuse Awareness Day 2023—AGE Platform Europe, 2023).

National prevalence studies also contribute to the illustration of the magnitude of elder abuse. In Australia, several studies have been conducted on that concern, with the National Elder Abuse Prevalence Study being one of the most representative ones, reporting that approximately 1 in 6 older adults had experienced at least one form of abuse, with psychological and financial abuse being the most common forms of elder abuse reported (Qu et al., 2021). Earlier studies similarly emphasized the widespread nature of elder abuse and stated significant barriers to disclosure and reporting, particularly in community settings (Kaspiew et al., 2016).

### 3.3. Risk Factors

Even though elder abuse should never be justifiable, it is important to understand the contexts in which it may occur in order to enhance prevention and intervention. The World Health Organization proposed a model by which the risk factors could be divided and analyzed: individual factors, relational factors, community factors, or social factors (Drager, 2002). These factors alone may not necessarily cause abuse, but most of the time, they lead to favorable circumstances that may attract it.

Individual factors are factors related to the victim. Often, functional dependency may be an important risk factor, as the elderly have difficulty performing daily activities such as eating, taking medication, or even personal hygiene (Rosen et al., 2020). The victim’s ability to recognize the abuse may be diminished in case of some cognitive impairment, thus leading to a higher risk of abuse (Wiglesworth et al., 2010). A study by Wiglesworth et al. estimated that the prevalence of abuse among elders suffering from dementia rises to about 47%, compared to 5–10% among the general population (Wiglesworth et al., 2010). Another risk factor is represented by social isolation, the most vulnerable being older people who do not get in touch often with family or with the community. In this context, victims are often unable or unwilling to report abuse due to fear of family conflict, social isolation, loss of contact with children, and increased dependency on caregivers. Evidence from official parliamentary reports suggests that many elders prioritize maintaining family relationships over seeking legal remedies, even when abuse is detected (Goiran et al., 2018). Most of the time, the abuse goes undetected due to the lack of witnesses (Acierno et al., 2010). In terms of gender, women are up to four times more susceptible to sexual abuse than men, and up to two times more susceptible to psychological abuse (Johannesen & Logiudice, 2013).

Relational factors are related to the relationships of the victim with family or caregivers. Abuse is more likely to happen if the situation between them becomes tense. A deficit in educational preparedness of the caregiver can lead to aggressive outbursts (Johannesen & Logiudice, 2013). Another important factor may be related to the financial status of the victim. There could be situations when the caregiver is financially dependent on the victim or simply wants to take advantage of the victim’s wealth. This kind of situation is associated with a higher risk of manipulation or emotional blackmail (Jackson & Hafemeister, 2013a). Particularly in financial contexts, situations perceived as abuse may arise from a variety of factors, including misunderstanding or over-involvement, while other cases represent intentional exploitation, illustrating the multifaceted nature of elder financial abuse (Victorian Parliament Law Reform Committee, 2010). Undiagnosed mental disorders, alcohol, or drug usage by the caregiver are also frequently associated with an increased risk of abuse (Lachs & Pillemer, 2015).

Social factors are more of a contemporary matter. Recent years have come with the appearance and rise of the phenomenon called “ageism”. It refers to perceiving the elders in a negative way due to the lack of productivity, regardless of other aspects such as wisdom and experience. This phenomenon could explain abusive treatment or a lack of intervention when abuse is noticed (Powell, 2018). Some studies also reveal that up to 30% of healthcare workers tend to have a negative perspective on elders (Nelson, 2002).

### 3.4. Elder Abuse in Community Settings

Abuse in community settings is a common type of abuse that often goes unnoticed. A study conducted by Yon et al. states that 1 in 6 elders has experienced at least one form of abuse in a community setting (Yon et al., 2017). The major difference between community and institutional abuse is relational context: in community settings, the victim is often abused by a close person (whether a family member, friend, or caregiver). This leads to an emotional bonding that may represent a psychological barrier to recognition or reporting the abuse. Global estimations are that only 1 in 24 cases of elder abuse are reported (Yon et al., 2017). The main characteristic of this kind of abuse is continuity—abuse tends to occur regularly, most of the time on a regular basis. For example, an older person may be exposed to neglect, humiliation, or financial restrictions by persons whom they consider to be close to them. Even though it may not generate visible signs of abuse, these situations leave a mark on the self-esteem and mental health of the victim (Lachs & Pillemer, 2015). There are also some kinds of situations when the abuse might be mistakenly perceived on the outside as “care” or “protection”, even though it represents the exact opposite. Some examples of this kind of abuse can be prohibiting contact with other people, medical decisions being made without consent, or financial manipulation. These situations represent a passive form of abuse that can easily go unnoticed and unreported (Storey, 2020).

Transactional abuse is also an aspect that should be taken into consideration. It refers to those specific cases in which the victim is conditioned to transfer some assets, sign some documents, or accept some compromises in order to get the needed support. The elderly person in this situation is at risk of being trapped in an abusive relationship, perceiving all of this like a “price” that they must pay in order to receive help and not be left alone or without resources (Storey, 2020). In extended families, the generational gap could be a real problem, as conflicts may arise over housing, inheritance, or the distribution of care responsibilities. All of these will eventually lead to emotional abuse or neglect of the elders. This is when “opportunistic caregivers” may arise—people who offer support to an elderly person in order to obtain some belongings, especially among single people (Roberto, 2016). The development of technology seemed to be an aid for older people with disabilities, or to those living alone, because it implied that they could seek professional help or easily report any form of mistreatment or abuse. However, another form of abuse may be related to taking control over the victim’s devices or digital accounts, thus monitoring phone calls, messages, or any means of trying to reach help (Bayne et al., 2023).

Besides reporting the abuse, preventing it should also be taken into consideration when it comes to elder abuse in community settings. This could be possible by engaging social workers, family doctors, or even volunteers to regularly get in contact with vulnerable people (Pillemer et al., 2016). Local authorities should put an emphasis on raising awareness among the population, with the purpose of a better understanding of the problem. The aim of these measures would also be to facilitate early recognition of risk situations (Sethi et al., 2011).

### 3.5. Elder Abuse in Institutional Settings

We define institutional settings as nursing homes, hospitals, or any other accommodation designed particularly for elder protection and care. Elder abuse in this kind of environment may come in any of the previously described forms of abuse, most of the time because the personnel are overworked, underpaid, or even unqualified for this kind of work. Neglect, intentional ignoring of requests, or not providing medical explanations are means of psychological abuse that tend to be normalized (Lachs & Pillemer, 2015). A survey addressed to elders living in institutional settings, done among 44 nursing homes, reveals that about 20% of elders experienced frequent verbal abuse from staff (Schiamberg et al., 2012). Thus, the most surprising aspect when it comes to abuse in institutional settings is not related to the nature of the aggression or form of abuse, but to the context, the abuse occurring in a specially designed environment in order to protect and support the elderly.

Unlike the community settings, where the aggressor could be easier to identify, abuse in institutional settings is harder to delimit, due to the collective nature. Some forms of mistreatment are more of a result of a faulty system, rather than of an inappropriate behaviour of the employee. Lack of privacy, inadequate spaces, forcing the elderly to eat at a fixed time, or the lack of free access to the bathroom are examples of structural abuses (Phelan, 2015). Living conditions can represent a form of abuse by themselves. Being forced to live in an overcrowded place, with decrepit furniture, or having denied access to hygiene or social activities can accelerate the physical and mental decline of the elderly (Post et al., 2010). Most of the time, in institutional settings, the victim is dependent on the aggressor, which may lead to the victim not reporting the abuse because of the fear of consequences, such as losing support for essential needs (medication, food, and personal hygiene). This kind of dependency leads to many abuses not being reported. The victims may even deny the abuse when asked directly (Cooper et al., 2009). A study conducted in Canada revealed that only 1 in 10 abuse cases has been officially reported (Cooper et al., 2009). Abusive behaviors often tend to become part of the organizational culture in the absence of real audits. Complicity could also be involved in some institutions. For example, even if an employee may detect an abuse, he could decide to cover it up, either because of fear of consequences or because of loyalty to the colleague (Phelan, 2015). Another form of possible abuse that could happen within an institutional setting is abuse between residents, consisting of intimidation or even aggression, whether physical or verbal, most of the time because of a lack of supervision (Castle, 2012). In a study published by N.G. Castle in 2012 in the *Journal of Elder Abuse & Neglect*, 21% of the interviewed staff admitted to at least one abuse case between residents in the past month (Castle, 2012).

### 3.6. Consequences of Elder Abuse

Physical consequences are related to the physical form of abuse. Its results are represented by injuries. The injuries may vary in severity, from bruises and fractures to internal trauma and even death. No injury should be underestimated because it has been demonstrated that, due to the physical decline of the elderly, even minor trauma can lead to loss of mobility (Dong, 2015). Neglect may be invisible at first, but over time, it has severe consequences on the health status. Dehydration, malnutrition, lack of hygiene, lack of medication administration, or neglecting some acute symptoms can lead to the worsening of the condition of the victim, producing irreversible complications (Strasser & Fulmer, 2007).

The consequences of psychological abuse may range from social withdrawal to even suicide attempts (Acierno et al., 2010). Constant stress, anxiety, or living in fear can be a trigger or aggravating factor for dementia (Wiglesworth et al., 2010). A study published by Wiglesworth et al. in the *Journal of the American Geriatrics Society* associates psychological abuse with a 30% higher risk of cognitive impairment in the following 5 years (Wiglesworth et al., 2010).

Social consequences are a result of isolation, stigmatization, and withdrawal of the elderly from social life. Being abused by a person with whom the elderly have an apparently close relationship or caregivers who are specially trained to protect them will lead to trust issues and further social withdrawal, meaning the elderly will get more and more isolated, thus affecting the chances of abuse being recognized or reported. Financial abuse also has some social consequences. There might be situations when an elderly person gets to a form of economic exclusion, not being able to access health services, to cover the basic needs, or they may also lose their housing. This kind of situation usually leads to prolonged institutionalization, not necessarily due to the health status, but more due to financial deprivation (Jackson & Hafemeister, 2013b). Financial abuse can generate severe situations in which the victim loses important assets and even housing, thus leading to social and economic vulnerability (Goiran, 2018).

Elder abuse also has some legal and ethical considerations. Most of the abuse cases can go unreported due to a lack of access to justice or fear of reprisals (Lachs & Pillemer, 2015). It is a common phenomenon for the victim to be unable or unwilling to report the abuse, giving up legal solutions in favor of maintaining good family relationships (Goiran et al., 2018). In the USA, only 1 in 14 abuse cases are being reported, according to a study from the National Research Council (National Research Council et al., 2002). Even when the cases are reported, most of them are quickly closed (Roberto, 2016). That usually happens because forensic evidence may be pretty difficult to obtain, especially when physical signs of abuse are missing or due to the presence of cognitive impairment in the victim. The ethical point of view of elder abuse states that society has a responsibility to care for its vulnerable members. Tolerating the abuse will only maintain suffering and inequality and represents a major social concern.

### 3.7. Forensic Aspects of Elder Abuse

Elder abuse can also be approached from a forensic point of view, due to its implications that extend beyond the clinical and social impact. The main forensic aspects of elder abuse consist of the importance of the healthcare professionals in providing legal support to the elderly and the challenges encountered in the recognition, documentation, and reporting processes. Healthcare professionals are frequently the first, and sometimes the only, individuals able to detect signs of abuse, particularly when victims are unable or unwilling to report the abuse by themselves. Previous studies have highlighted that inadequate recognition, documentation, and reporting of the abuse contribute to underreporting and limited access to legal protection for the victims (Cooper et al., 2009; Lachs & Pillemer, 2015). Moreover, comorbidities and age-related fragility may interfere in a proper differentiation between accidental and intentional injuries, emphasizing the role of the forensic perspective in the assessment of elder abuse (LoFaso & Rosen, 2014).

The role of the physician in detecting and reporting abuse has been increasingly emphasized in recent times. Most of the time, the physician (whether a family physician, an emergency physician, or a specialist) is the first person to get in contact with the victim. Besides the therapeutic responsibility, the doctor also has the legal responsibility to suspect, detect, and report any form of abuse the elderly might be exposed to, even when the victim doesn’t admit it. In Romania, the Medical Code of Ethics supports the role of the doctor’s involvement in this kind of situation (Colegiul Medicilor din România, 2025). Even though some injuries may be mistakenly perceived as accidental trauma, due to balance disorders in the elderly, making it difficult to identify the abuse, some aspects, such as incongruence between the injuries and the victim’s statements or injuries in areas that are not normally exposed, should be further investigated (Lachs & Pillemer, 2015).

When it comes to forensic expertise, the physician has a huge role in identifying and investigating injuries and establishing the cause-and-effect relationship between the trauma and its medical effects. The physician should take into consideration some aspects while investigating an abuse: Complete medical history; the victim’s comorbidities that may interfere with the lesions (like a hematoma, which can be a cause of physical abuse, but also can be caused by anticoagulant medication); and the victim’s biological features (e.g., thin skin, osteoporosis) (LoFaso & Rosen, 2014). In case of cognitive impairment, a multidisciplinary assessment of the situation must be approached, involving neurologists, psychiatrists, or geriatricians, because the victim’s ability to provide consent and to express their experience is essential for a proper forensic classification.

A proper documentation of the injuries and the context in which they appeared is important in supporting the case. Monitoring the injuries over time by taking successive photographs, paying attention to the localization, changes in size, and color, is essential for proper documentation. The victim’s statements are also important, so any statement related to the topic should be recorded at any moment. The context, the statements, and the injuries should be clearly described in the forensic certificate, mentioning not only physical trauma but also the signs of neglect (bedsores, dehydration, poor hygiene) (Fulmer, 2008). A rigorous documentation and description of the matter is crucial, as the entire process may be at risk of being compromised in the absence of it.

Forensic documentation implies some legal and ethical challenges. Despite being victims of abuse, most of the elders tend to be reticent about it either out of loyalty to the aggressor (often in community settings, represented by a family member) or out of fear of repercussions (in institutional settings, due to the total dependence of the victim to the abuser, most of the times the caregiver), thus jeopardizing the entire process. Cognitive impairment also implies some challenges in documenting the abuse, due to the inability to perceive the abuse or understand its implications. If it is proven that the elder is not able to represent their interests, social services can be involved. Thus, legal or medical decisions will be made by the latter, according to the principles of bioethics, seeking the best interests of the patient (Donovan & Regehr, 2010).

## 4. Discussions

In this review, elder abuse is presented as a multidimensional issue. The results of this review confirm that elder abuse is frequently present as different forms overlapping each other (physical, psychological, financial, sexual abuse, and neglect), rather than one singular form of abuse. Different forms of abuse have different risk factors and consequences, but the common aspect is that they still tend to strengthen one another, with effects varying from immediate injury to prolonged psychological, social, and legal consequences. These characteristics may explain why elder abuse still remains difficult to detect and is not adequately addressed worldwide.

A large discrepancy between reported prevalence and actual occurrence is evident, particularly in community settings. Studies in that direction indicate that only a small number of cases were officially reported, not only due to lack of awareness, but mostly due to structural and relational factors, such as emotional dependence on the aggressor (often being a family member), fear of consequences, or the normalization of abusive behaviors within the family (Acierno et al., 2010; Yon et al., 2017). Thus, the elders are prone to chronic exposure, which reinforces their vulnerability.

Two particular forms of abuse that often tend to overlap are psychological abuse and financial exploitation. Gaslighting, emotional manipulation, and coercive control precede or accompany financial abuse, gradually undermining the older person’s autonomy and perception of reality. International reports and legal inquiries emphasize the frequent association of financial abuse with dynamics of emotional domination, rather than occurring as an isolated act (Goiran et al., 2018).

From a medico-legal perspective, the findings of this review emphasize the role of healthcare professionals in reducing the gap between the health and justice systems. Most of the time, the physician is the first, and even only professional who interferes with abused older adults. Comorbidities and age-related fragility may complicate the differentiation between accidental and intentional injuries, increasing the risk that abuse will be dismissed (LoFaso & Rosen, 2014). Vague clinical language, failure to recognize particular patterns of injury or neglect, or inadequate documentation may diminish the possibility of legal action (Cooper et al., 2009). Furthermore, the absence of standardized protocols for screening, documentation, and forensic assessment contributes to inconsistent clinical practices and unequal levels of protection for older adults. In this context, the physician may have doubts about his legal and ethical obligations, particularly in settings where mandatory reporting requirements are poorly defined (Dev & Eljo, 2024).

Finally, this review identifies important gaps in the existing literature. Even though prevalence and risk factors have been widely studied, considerably fewer studies examine how clinical findings translate into legal action or assess the impact of medico-legal interventions. A more precise operational definitions, enhanced training of healthcare professionals, and research focusing on outcomes of reporting, forensic assessment, and legal involvement are required. Increased awareness is not enough in addressing elder abuse, but also structural changes that support early detection, ethical clinical practice, and meaningful access to justice.

## 5. Conclusions

The use of the term “elder abuse” when discussing the gap between care and protection for older people is a current issue in modern medicine. This paper presents a multidimensional view of the concept of elder abuse (i.e., physical abuse; emotional/psychological abuse; financial abuse; sexual abuse; neglect) and its dimensions, and how these abuses can occur concurrently, independently, and cumulatively, leading to a variety of outcomes including increased risk of morbidity, psychological distress, social isolation, and a loss of autonomy among older adults.

Therefore, it is essential to consider both societal and professional factors when developing an approach to reduce the negative consequences of elder abuse. Societal approaches must address and challenge ageist attitudes towards older people to eliminate the perceived acceptability of abuse of older people. Therefore, elder abuse must be recognized as a preventable form of violence that has serious health and legal consequences. Professional approaches require the development of standardized procedures for identifying, documenting, and reporting elder abuse in health care settings. The inclusion of clinical, ethical, and medico-legal competencies in professional education/training will enable professionals to identify early signs of elder abuse, differentiate between accidental and intentional harm, and provide consistent professional decisions regarding the treatment of older adults who have been abused.

Finally, greater legal protection for vulnerable elders and improved access to forensic evaluations are necessary to reduce the number of cases of elder abuse that go unreported. This review provides a multidisciplinary perspective on elder abuse by recognizing elder abuse as both a medical problem and a justice issue, highlighting the need for interdisciplinary approaches to promote safety and dignity for older adults in our aging society.

## Figures and Tables

**Table 1:** A summary of different forms of Elder Abuse.

Type of Abuse	Definition	Indicators	Reference No.
Physical	Use of violence to provoke pain, injuries, or suffering upon an elder person	Bruises, fractures, abrasions, lacerations, or burns in atypical locations	(Dong, 2015; Lachs & Pillemer, 2015)
Psychological	Verbal or nonverbal acts that cause emotional distress	Low self-esteem, anxiety, or even depression	(Roberto, 2016; Wiglesworth et al., 2010)
Financial	Use of an elderly person’s financial resources or assets in an illegal, unethical, or unauthorized way	Missing funds, suspicious transactions, coerced signatures	(Jackson & Hafemeister, 2013a)
Neglect	Failure to provide essential care (nutrition, hygiene, medication)	Malnutrition, dehydration, decubitus ulcers, infections	(Lachs & Pillemer, 2015)
Sexual	Non-consensual sexual contact or exposure	Bruises, abrasions, lacerations in the anogenital area or abdomen, even UTIs	(Bows, 2018; Teaster & Roberto, 2004)

**Table 2:** Prevalence of Elder Abuse by setting (based on data from (Yon et al., 2017, 2019)).

Type of Abuse	Community Prevalence (%)	Institutional Prevalence (%)
Physical	6.8%	14.1%
Psychological	11.6%	33.4%
Financial	6.8%	13.8%
Neglect	4.2%	11.6%
Sexual	0.9%	1.9%
Overall	15.7%	~64% (staff self-reported)

## Data Availability

No new data were created.
